# Exploring the viewpoint of faculty members of medical sciences universities about effective factors on their organizational retention: a qualitative study

**DOI:** 10.1186/s12909-023-04707-1

**Published:** 2023-10-03

**Authors:** Elaheh Mohammadi, Mahla Salajegheh

**Affiliations:** 1 Health Professions Education Research Center, Educational Development Center, Tehran University of Medical Sciences, Tehran, Iran; 2 Department of Medical Education, Medical education development center, Kerman University of Medical Sciences, Kerman, Iran

**Keywords:** Organizational Retention, Faculty, Faculty’s retention, Medical Sciences, Human resource, Organizational loyalty

## Abstract

**Background and aim:**

Faculty retention in medical sciences universities is one of the most important values for the survival of the educational system. This study aimed to explore the viewpoint of faculty members of medical sciences universities in Iran about effective factors affecting their organizational retention.

**Methods:**

Qualitative study using deep interviews collected by maximum variation sampling. A purposively selected sample of 15 faculty members was recruited throughout two Iranian medical sciences universities (Tehran University of Medical Sciences and Kerman University of Medical Sciences) during 2021-22.

**Results:**

Qualitative data analysis provided 3 main categories and 10 sub-categories. Three main categories were identified that affected the faculty’s organizational retention included individual factors, institutional factors, and socio-political factors.

**Conclusion:**

Factors that contribute to the improvement of faculty retention encompass various aspects. These include consideration given to the personal and social requirements of faculty members, support provided by the organization along with effective resource management, a fair and transparent organizational structure, and the maintenance of political stability both within the university and the nation.

## Introduction

Nowadays, efficient human resources are considered the most valuable asset of any organization and play a significant role in productivity [1, 2]. Organizational retention refers to a process in which human resources are encouraged in different ways to remain in an organization for a maximum period of time [3]. This causes the members of an organization to use all their strengths to achieve the goals of the organization and work enthusiastically toward them [4]. Organizations incur a lot of costs in hiring and developing employees and enhancing their skills, so the departure of elite and experienced forces causes a waste of these valuable resources [2].

To provide healthcare services to society as well as to educate students, medical universities require the hiring of motivated and efficient faculty members since they are the most important human resources in the process of improving the quality of education and health [5, 6]. After hiring faculty members, medical universities make a significant investment in their development and empowerment. So, their premature departure is very costly for the universities, making the retention of these forces in medical universities very important [7]. However, some of these faculty tend to leave the medical sciences university, due to personal and organizational reasons.

Despite the significance of this topic, limited research has examined faculty retention in medical sciences universities and the variables influencing it. Marchiori et al. (2004) identified tenure in higher education, gender, and age as factors affecting the organizational commitment of health profession faculties in the United States and Canada in their study on the dimensions and conditions of the organizational commitment of health profession faculties [8]. Radafshar et al. (2010) have investigated the motivation factors affecting the retention of 71 faculty members at one medical sciences university in Iran and indicated that factors such as climate, healthcare, and entertainment facilities of the city, the possibility of working in the private sector, and having good financial condition influence the retention of faculty members [9]. In their study on faculty retention in private higher education institutions in Pakistan, Mubarak et al. (2012) concluded that the variables “pay satisfaction” and “learning and growth opportunities” significantly influence faculty retention [10]. Xierali et al. (2021) found that in one of the American medical schools, the 10-year retention rate for 4279 assistant professors was only 43% [11]. In Iran, about 400 general doctors and 300 to 350 specialist doctors emigrate every year, and about 180,000 educated Iranians leave the country each year in hopes of finding a better life and job opportunities [12]. So exploring the effective factors on faculty organizational retention is essential since it helps to gain more knowledge about the needs of faculty members and can be useful to motivate them to retain longer in the system. The present study aimed to explore the viewpoint of faculty members of medical sciences universities in Iran about effective factors affecting their organizational retention.

## Methods

### Design

A qualitative design using conventional content analysis was performed. A qualitative content analysis method allows the deep exploration of experience as well as the interpretation of the data, leading to conclusions about the meaning of these experiences [13].

### Setting

Between September 2021 and July 2022, this study was carried out at the Kerman and Tehran Universities of Medical Sciences. It was attempted to choose universities for sampling from two different zones, which express the individual differences of Iranian medical sciences faculty members as much as possible, taking into account the geographical extent of Iran and the fact that this country’s medical sciences universities are divided into 10 zones.

### Participants

The population included faculty members affiliated with Tehran University of Medical Sciences and Kerman University of Medical Sciences. From those eligible for inclusion, 15 (11 female) faculty were purposefully selected through snowball sampling. Almost half were affiliated with Kerman University of Medical Sciences (N = 8). The majority of participants were assistant professors (N = 10), 4 were associate professors, and the rest were instructors. Almost half of the faculty were affiliated with clinical science departments (N = 7). The inclusion criteria were being interested in participating in the study and having at least 6 months of experience as a faculty member at Tehran or Kerman Universities of Medical Sciences. The exclusion criteria were unwillingness to participate in the study, non-medical science faculty, less than 6 months of experience as a faculty member, unwillingness to conduct or continue the interview.

### Data collection

Firstly, questions and an interview guide were compiled from a literature review. The questions were as follows:

- Please describe your expectations as a faculty member from the university.
- Based on your experience, what are the factors that led to organizational loyalty as a faculty member?
- From your lens, what factors can lead a faculty to leave the organization?
- Have you ever intended to leave the university? What factors caused you to think this way? What factors made you not leave the organization?

Then 15 in-depth interviews with a duration of approximately one hour, were conducted and transcribed verbatim.

### Data analysis

The traditional qualitative content analysis method was used to assess the data [14]. For improved comprehension and overall comprehension, the initial author (EM) read the transcripts numerous times. Then, units were separated out of each transcript. The meaning units were classified, and depending on similarity and difference, the codes were further broken down into sub-categories and categories.

### Rigor

Each of the analytic reports was reviewed in terms of reflecting the viewpoints of the participants. The research team reflected on the findings of the study and reached a consensus. A summary of the transcripts was returned to the participants as a member check and approved by them. A peer check was also performed by one colleague who was familiar with the qualitative content analysis method. To confirm dependability, several transcripts were randomly analyzed by an audit to ensure the accuracy of the process. The plausibility of the findings confirmed that the analyses and interpretations were justifiable. To confirm reflexivity, the extracted codes and categories were agreed upon by the research team [15].

### Ethical considerations

The Ethical Review Board of the National Agency for Strategic Research in Medical Education approved the study (IR.NASRME.REC.1400.334). Participants did not receive any incentives, and participation was voluntary. Informed verbal and written consent for participation was obtained based on the proposal approved by the ethics committee. The participants were also assured of the confidentiality of their information, and it was explained that the results would only be used for research objectives.

## Results

15 faculty members participated in this study. The demographic characteristics are available in Table 1.

**Table 1:** The demographic characteristics of the participants

University of Medical Sciences	Gender		Clinical or Basic science		Academic Rank			Total
	Female	Male	Clinical science	Basic science	Assistant professors	Associate professors	Instructor	
Tehran	5	2	4	3	5	2		7
Kerman	6	2	3	5	6	1	1	8

Qualitative data analysis provided 3 main categories and 10 sub-categories. The three categories included individual factors, institutional factors, and socio-political factors. (Table 2)

**Table 2:** the results of data analysis

Main categories	Sub-categories	Codes
Individual factors	Faculty members’ needs	Dignity and position of faculty in university
		Academic needs
		Personal needs
		Financial needs
	Personal interests, motivations, and beliefs	Interest in education
		Interest in serving the country
		Giving importance to the prestige and position of university
		Importance of social status of faculty
	Personality traits	Patience
		Contentment
		Resilience
		Risk-Taking
		Dependence
	Family issues	
Institutional factors	Organizational structure and culture	Organizational support
		Justice
		Transparency in the organization
		Organizational management approach
		Faculty participation in decision-making
		Possibility of professional growth and development
		Atmosphere governing human relations in university
		Resource management
		Job security
	Faculty valuation system	
	Facilities and equipment available in university	Facilities and equipment for educational
		Facilities and equipment for research
		Welfare affairs
Political factors	Political stability in country	
	Political stability at university	
	Sociopolitical concerns of society	

### 1. Individual factors

The “individual factors” category included 4 subcategories, including (1) Faculty members’ needs, (2) Personal interests, motivations, and beliefs, (3) Personality traits, and (4) Family issues.

### Faculty members’ needs

The participants discussed intellectual, personal, and financial demands as well as faculty members’ dignity and status within the institution as crucial elements determining faculty retention in the organization. Some of the people interviewed believed that behaviors and attitudes that were beneath a faculty member’s dignity were causes of faculty turnover.

*“I frequently see no respect. For example, there is no place for me to change my clothes or put my things in the hospital. This is one of aspect of respect. Rude behaviors are frequently observed in the system. All of these gradually make one think, what is his position?“* (P10).

All the interviewees referred to financial needs as a factor affecting faculty retention at the university.

“*Nowadays, the matter is not low or high income. The question is whether the income is adequate for me to be able to satisfy my basic needs or not. And many times, it’s not adequate. Obviously, if you are not able to meet your basic needs, you wouldn’t work well.*“ (P13).

### Personal interests, motivations, and beliefs

The importance of the social status of the faculty member is mentioned as one of the most significant factors affecting faculty retention in the organization, along with interest in education, a desire to serve their country, and the importance given to the standing and prestige of the university where they work.

“*I like teaching. It’s enjoyable for me. I study and update my knowledge. The reasons for faculty turnover are burnout and a lack of interest. Although being a good doctor is important, being a good teacher is more important. It provides a higher level of prestige and higher social* status.“ (P2).

### Personality traits

Personality traits, as inseparable factors of human personality, significantly affect employee retention in the organization [4]. Some professors believed that character attributes like dependability, resilience, patience, and satisfaction were useful in keeping students at the university where they taught.

“*Individuals’ personalities play a key role in their decisions. Their resilience and patience make them stay. I grew up in a family where I learned to be content. Family culture is important. Thank God, my father helped me to buy an apartment, so financial issues don’t bother me too much so that I want to quit being a faculty member*” (P12).

### Family issues

Another factor that can be considered effective in faculty retention is the issue of to the faculty member’s family.

Sometimes faculty members intend to leave the organization because of their family’s issues, problems, or preferences, while they may be satisfied with their job conditions in the organization. Or, on the contrary, they want to leave the organization, but family issues do not allow them to do so. Therefore, one of the factors that plays a role in the longevity of faculty members is family interest.

“*It is mostly because of the atmosphere governing society, not the university itself that faculty members decide to leave the university. They emigrate because of the family conditions and their concern for the future of their children. They want to provide better conditions for their children …*” (P4).

“*Many people stay at the university because of family issues. For example, they have parents to take care of, so they prefer to stay at the university; otherwise, they have to go to other cities. Or they have a child, and this makes it impossible for them to change or emigrate*” (P4).

### 2. Institutional factors

A participant’s retention in the organization is reportedly impacted by a number of factors, including those connected to the university where they work. The organizational structure and culture, the mechanism for evaluating teachers, and the facilities and equipment offered by the university are the three subcategories that make up this category.

### Organizational structure and culture

The structure and culture of any organization play a decisive role in the motivation and desire of people to stay in the organization. According to the participants, factors related to organizational structure and culture such as organizational support, justice, transparency in the organization, organizational management approach, faculty participation in decision-making, the possibility of professional growth and development, the atmosphere governing human relations in the university, resource management, and job security play an effective role in the faculty members’ desire to stay at the university where they work.

“*Whoever enters the university system expects support from the organization, but there is no support. For example, I want to do research now, but there are limited databases available at the university. So, there is no difference between being and not being in this academic environment. Or, for example, we wanted to participate*: *but academic correspondence took so much time that the visa appointment was lost. It’s disappointing*” (P6).

Regarding transparency in the organization, another participant believed that there was poor transparency in the organization:

“*Many times, it seems to me that I have no participation in the programs, and I do not understand what decisions are made on what basis. These issues cause pessimism in people, often making young faculty leave the university and emigrate*” (P7).

### Faculty evaluation system

Some of the interviewees considered faculty evaluation a factor influencing their retention in the organization. According to them, although the most important goal of faculty evaluation is educational improvement, sometimes the results of this evaluation are a factor causing faculty members to not stay at the university where they work.

“*There is no congruence between the real educational practice and the evaluation method considering the high workload and executive tasks*” (P2).

### Facilities and equipment available in the university

Some faculty believed that the lack of necessary facilities and equipment for educational, research, and welfare affairs was one of the obstacles to faculty retention in the organization.

“*Sometimes I type something when I visit a patient. A few days ago, when I was working at a private hospital, I found that my keyboard had a problem. I asked the relevant official to replace it, and he/she did it immediately, but at the public hospital where I work, there is a computer that has been problematic for a month and we have frequently asked to replace or repair it, but no measure has been taken. This shows that the system is not worth working for”* (P9).

### 3. Political-social factors

The factors in the socio-political context of the universities and also the country are very effective in faculty retention. This category includes three sub-categories of political stability in the country, political stability at the university, and sociopolitical concerns in society.

### Political stability in the country

Political stability refers to the presence of a peaceful and reliable sociopolitical atmosphere and the continuity of laws, management, and policies [16]. According to the experiences of several participants, to reach political, social, and economic development, faculty members, as the most important pillar playing a role in promoting the quality of education in the country, require that the conditions in the country and at the universities ensure a certain level of stability to make it possible for them to plan and follow development goals.

“*Changes in the policies of the country in different periods cause changes in the programs and administrations of the ministry, which in turn lead to changes in the prevailing procedures and approaches in the university*” (P7).

### Political stability at the university

It is important to separate the scientific issues of the university from the politics of the country. So, that it is not necessary to change the management of the university with every political change in the country. These changes sometimes cause delays in the current affairs of the university and change the procedures for the academic approaches of faculty members, which can lead to discouragement of them.

“*The political changes happening in the university do not allow you to do some things. To do this, you have to show and prove yourself to some people. This is time-consuming and will take me four years to do it, and those to whom you are trying to prove yourself will be changed. This greatly influences your motivation to stay or leave”* (P3).

### Sociopolitical concerns of the society

The social and political crises governing society play a key role in achieving the development of society by affecting the motivation and efforts of faculty members in the arena of social and political evolution.

*“During a period when there was more hope for progress, fewer faculty emigrated, and even many of my friends returned to Iran after finding that the conditions were ready for progress. If society is progressing, organizational ties will be formed”* (P8).

## Discussion

Considering the importance of faculty members of medical sciences universities as social assets of the country, it is important to study their views on the factors affecting their organizational retention. The present study aimed to explore the viewpoint of faculty members of medical sciences universities in Iran about effective factors on their organizational retention.

According to the results of the present study, one of the most important factors affecting faculty retention at medical sciences universities is individual factors. Faculty members have a series of personal, academic, and financial needs, and their fulfillment plays an important role in the satisfaction and longevity of faculty members in universities.

The results of previous studies indicate the positive effect of the support and respect received from the organization on the faculty members’ commitment and loyalty to the institution where they work (17, 18). Khanipour et al. (2019) investigated perceived social status and its organizational, managerial, and political predictors among Iranian faculty members. They reported that participants believed that the social status of faculty members had decreased (19). Given that social status is regarded as a psychosocial process that could be efficient for faculty members’ scientific and professional lives, this finding revealed that an appropriate position leads to more longevity in the university.

Faculty members must stay current and pick up knowledge and skills to keep up with global scientific advancement. Our findings highlighted the faculty members’ belief that the institution must give them access to the best and most effective training programs needed both domestically and internationally. As a result, one of the requirements in the academic environment is to plan for implementing beneficial empowerment programs and carrying out educational interventions to enhance the educational experiences of the faculty members and motivate them to participate in these programs [20]. Faculty development evaluation shows their desire and effort to be up-to-date in the field of medical education and to know various fields of medical education [21].

Regarding financial needs, the importance of income has been emphasized as one of the main factors influencing faculty retention in organizations (22). Faculty members who receive higher salaries show a higher level of retention in their institution compared to their colleagues who receive a lower salary (23). The present results emphasize that increasing the faculty members’ salaries and responding to their financial needs are related to their increased retention in the organization. Notably, all the participants of different academic ranks at both Tehran and Kerman Universities of Medical Sciences greatly highlighted the financial issue and considered it one of the main concerns of the faculty members. The participants in this research believed that their present income was not adequate to meet their needs considering their families and society’s expectations of a faculty member. The lack of coordination between the faculty members’ personal and professional values and the management policies of the organization where they work, plays a significant role in the job dissatisfaction of faculty members and their desire to leave the university [24, 25].

The personality traits of faculty members are among the factors influencing their retention in the organization. In similar situations, individuals may make different decisions according to their personality traits. Bandura’s social-cognitive theory of human functioning is one of the most widely used theories applied to predict and understand behaviors. This theory emphasizes that individual and environmental characteristics affect an individual’s behavior (23). As a result, faculty members’ organizational loyalty may be influenced by their personal traits, such as dependability, resilience, and dependability, which were retrieved in the present. The findings show that deontological individuals have stronger intrinsic drives. These individuals frequently have a tendency to stay in the firm and carry on working despite all of the current issues. Another person can have similar circumstances but still want to leave the company because of their unique personalities. In fact, those with greater resilience are able to deal with adversity better and are less likely to want to leave the company due to ongoing issues. Previous research has found a connection between resilience and the decision to remain employed by an educational institution. So, it is possible to improve organizational retention by implementing appropriate interventions and this relationship can be used for the psychological development of faculty. The high resilience of faculty members provides the groundwork for their successful encounters with challenging situations [26]. Having social support, such as the possibility of interaction with colleagues and organizational administrators, can enhance resilience in the educational institution environment [27].

Employee turnover requires high risk-taking. People who hardly manage changes and do not have a risk-taking personality are usually not willing to change their work environment easily. Despite the importance of faculty retention, there are limited studies on how demographic variables and professional life issues are related to job satisfaction, loyalty to the organization, and the desire to stay in it (26). However, the findings highlight that faculty members’ perceptions of the relationship between their work life and risk-taking have a powerful direct effect on satisfaction and, thereby, the intention to leave the organization [28].

Family issues are one of the most important factors affecting the retention of faculty members. The ability of the university as a workplace to meet the faculty members’ needs to improve family life conditions is an important concept related to faculty retention in the organization [29]. Also, social conditions affecting the welfare of the family and the future of children play an effective role in faculty retention. According to the results of the present study, family issues can be a factor in both faculty retention, despite their inner desire, and turnover and even emigration. The participants in the present study believed that they are sometimes forced to continue their work at the university because of the duties they have towards their parents or the presence of their young children, and sometimes, they want to leave the university and emigrate because of their worries about the future of their children.

According to participants, factors related to the university as their workplace, including the organizational structure and culture, the faculty evaluation system, and the available facilities and equipment, are among the factors affecting faculty members at medical sciences universities. Faculty members in institutions with collectivist organizational cultures and collaborative leadership have higher spirits and job satisfaction (30). Küskü (2003) investigated the job satisfaction of faculty members in Turkey and reported that faculty were less satisfied with university administrators than their administrative colleagues (31). This finding shows that faculty members have higher expectations of university administrators. Similarly, Schroder (2008) found that fewer faculty members with PhD degrees were less satisfied with their institution’s management compared to their colleagues with MSc degrees [32]. Also, Bibi et al. (2018) showed that faculty development programs directly influence faculty retention [33]. These studies confirm that faculty members consider the management approach and the prevailing organizational culture of involving individuals in academic decision-making, and organizational support of faculty members, respect for them, and their involvement in decision-making positively influence their organizational commitment [32–34].

Our results revealed that socio-political factors are another factor affecting faculty retention at medical sciences universities. These findings indicate the characteristics that affect the individual through the political atmosphere that governs the university and the country. These controversial concepts deal with the perceptions of the faculty members about the policies of the university and the country, and it shows that faculty consider the country and their organization as political work environments in nature. Even Khanipour et al. (2019) revealed that organizational politics could predict perceived social status [19], which we found to be one of the most important factors affecting faculty retention at medical sciences universities. Of course, there is a lack of evidence in the field of the relationship between political stability in the country or in the university and organizational retention of faculty members.

Moreover, the participants who were affiliated with Tehran University of Medical Sciences believed that socio-political factors played a more significant role among the factors influencing their retention in the organization, while the faculty who were affiliated with Kerman University of Medical Sciences assumed that justice in the division of responsibility and executive affairs were more important. This result can be justified considering the location of Tehran University of Medical Sciences in the capital, which makes it face more political issues. While at Kerman University of Medical Sciences, which is located at a greater geographical distance from the capital, all interviewees from this university agreed on the more significant role of organizational justice and fair distribution of burdens and benefits in their retention. Madurani and colleagues (2022) revealed that there is a direct consequence of talent management on organizational member retention and an indirect effect mediated by organizational justice [35].

Due to the limitation of sampling in this study, it should be noted that the topics mentioned above were the result of interviews with only 15 faculty members.

### Limitations

Firstly, in this study we only investigated the viewpoints of faculty members, but it seems that it is necessary to know the views of other stakeholders in the medical education system, such as human resources managers and policymakers to enhance the richness of the data. Therefore, it is recommended to examine the views of other stakeholders in future research. Secondly, in this research, 15 faculty members were interviewed. A larger sample size that is balanced in terms of gender and addresses specific questions in clinical and basic sciences separately would be helpful. Thirdly, the interviews were conducted in only two universities in the country, which were chosen due to the researchers’ easier access to the samples. Fourthly, conducting interviews with a small sample size cannot comprehensively confirm the weaknesses of faculty members retention in Iran. Therefore, it is recommended to future studies to perform mix method researches in this regard.

### **Suggestions for future studies**

We recommended that future research take into account more samples, since this would increase the generalizability of the results. The research’s interview questions, which mainly focused on the devotion of faculty members to the organization, revealed that neither the clinical and basic sciences faculty nor the faculty’s gender yielded any distinct themes that could be separated. In order to better understand the perspectives of clinical and fundamental science faculty members as well as professors who are male or female, it may be good for future studies to ask different questions.

## Conclusion

According to the results of the present study, individual factors, institutional factors, and socio-political factors are effective in the influencing faculty retention in medical sciences universities. As factors playing a role in enhancing faculty retention, as the foundation of medical universities, one can refer to the attention paid to the faculty members’ individual and social needs, organizational support and proper resource management, transparency and justice in the organization, as well as the political stability in the university and the country. Human resource planners in medical sciences universities should consider individual factors, institutional factors, and socio-political factors that affect the faculty’s organizational loyalty. As a conclusion, providing financial needs, keeping the prestige of faculty members, and involving them in decision-making and meritocracy in the management positions of the university can be effective as practical suggestions for the organizational retention of faculty members in medical sciences universities.

## Data Availability

The datasets used and/or analyzed during the current study are available from the corresponding author on reasonable request.
